# Clinical Nomogram to Predict Major Adverse Cardiac Events in Acute Myocardial Infarction Patients within 1 Year of Percutaneous Coronary Intervention

**DOI:** 10.1155/2021/3758320

**Published:** 2021-12-13

**Authors:** Defeng Pan, Shengjue Xiao, Yue Hu, Qinyuan Pan, Qi Wu, Xiaotong Wang, Qiaozhi Liu, Ailin Liu, Jie Liu, Hong Zhu, Yufei Zhou

**Affiliations:** ^1^Department of Cardiology, The Affiliated Hospital of Xuzhou Medical University, Xuzhou, Jiangsu 221004, China; ^2^Department of General Practice, The Affiliated Hospital of Xuzhou Medical University, Xuzhou, Jiangsu 221004, China; ^3^Shanghai Institute of Cardiovascular Diseases, Zhongshan Hospital and Institutes of Biomedical Sciences, Fudan University, Shanghai 200032, China

## Abstract

The purpose of this study was to summarize the clinical characteristics and risk factors of major adverse cardiovascular events (MACEs) in patients who had had acute myocardial infarction (AMI) within 1 year of percutaneous coronary intervention (PCI). A total of 421 AMI patients who were treated with PCI and experienced MACEs within 1 year of their admission were included in this retrospective study. In addition, patients were matched for age, sex, and presentation with 561 patients after AMI who had not had MACEs. The clinical characteristics and risk factors for MACEs within 1 year in AMI patients were investigated, to develop a nomogram for MACEs based on univariate and multivariate analyses. The *C* statistic was used to assess the discriminative performance of the nomogram. In addition, calibration curve and decision curve analyses were conducted to validate the calibration performance and utility, respectively, of the nomogram. After univariate and multivariate analyses, a nomogram was constructed based on age (odds ratio (OR): 1.030; 95% confidence interval (CI): 1.014–1.047), diabetes mellitus (OR: 1.667; 95% CI: 1.151–2.415), low-density lipoprotein cholesterol (OR: 1.332; 95% CI: 1.134–1.565), uric acid (OR: 1.003; 95% CI: 1.001–1.005), lipoprotein (a) (OR: 1.003; 95% CI: 1.002–1.003), left ventricular ejection fraction (OR: 0.929; 95% CI: 0.905–0.954), Syntax score (OR: 1.075; 95% CI: 1.053–1.097), and hypersensitive troponin T (OR: 1.002; 95% CI: 1.002–1.003). The *C* statistic was 0.814. The calibration curve showed good concordance of the nomogram, while decision curve analysis demonstrated satisfactory positive net benefits. We developed a convenient, practical, and effective prediction model for predicting MACEs in AMI patients within 1 year of PCI. To ensure generalizability, this model requires external validation.

## 1. Introduction

It is widely recognized that acute myocardial infarction (AMI) is the disease with the highest morbidity and mortality worldwide; the most common cause of AMI is local thrombosis caused by ruptured atherosclerotic plaques [1]. Numerous studies have revealed that primary percutaneous coronary intervention (PCI) can increase myocardial blood flow perfusion and improve clinical outcomes [2]. However, AMI patients who are undergoing PCI still have an increased risk of recurrent major adverse cardiovascular events (MACEs) [3]. This is because the pathogenesis of AMI is complex. Previous research conducted with patients in Western countries has demonstrated that diabetes, current smoking, history of heart failure, and high Syntax score are independent prognostic factors of MACEs for AMI patients who are treated using PCI [4, 5]. However, there has been little research to explore the cardiovascular risk stratification and outcome prediction in Chinese AMI patients after successful PCI.

Nomograms are widely used to predict disease prognosis for individual patients. Recently, a significant number of novel models have been employed to predict clinical outcomes following PCI, but these are still far from ideal [6]. Some studies have shown that detection of the serum myocardial enzyme spectrum plays an important role in indicating myocardial injury and can judge the severity of disease, and some myocardial markers, such as cardiac troponin, can accurately reflect the situation of myocardial injury [7, 8]. In addition, bile acid as a risk factor and predictor of cardiovascular disease has gradually attracted people's attention. Studies have shown that elevated serum levels of bile acid are associated with the development of coronary heart disease [9]. However, it is not yet clear how to judge the prognosis of AMI patients accurately. Thus, our study is aimed at establishing a prognostic nomogram based on clinical parameters for use with Chinese patients that can help clinicians to both identify high-risk patients and provide them with appropriate treatment through a more accurate risk assessment.

## 2. Material and Methods

### 2.1. Study Population

All AMI patients who were older than 18 years and had undergone PCI at the Affiliated Hospital of Xuzhou Medical University between January 2015 and December 2020 were screened for inclusion. The third universal definition of myocardial infarction [10] was used to define AMI. Inclusion criteria were as follows: (1) Patients were diagnosed with either ST-segment elevation myocardial infarction (STEMI) or non-STEMI (NSTEMI). (2) Coronary angiography revealed that patients had received PCI for anatomy in the infarct-related artery. (3) The follow-up time was at least 12 months. Patients were excluded from the study if they: (1) lacked baseline or follow-up data, (2) had other heart disease or malignant tumors, and (3) had a previous history of acute coronary syndrome. Supplementary File S1 gives the detailed inclusion and exclusion criteria for AMI patients. All participants completed the questionnaire to assess their eligibility for clinical research. Patients provided their informed consent to take part in the study; the study questionnaire is given in Supplementary File S2.

### 2.2. Baseline Data Collection, Periprocedural Medications, and Blood Sampling

Clinical data were systematically collected at enrolment and at 1 year after index admission from electronic medical records. Data included demographic details, clinical characteristics, risk factors, and medications. Adequate dual antiplatelet therapy was defined as chronic treatment with aspirin and a P2Y12 receptor antagonist (clopidogrel or ticagrelor), or aspirin loading at least 2 h prior to recruitment with either clopidogrel at least 6 h or ticagrelor at least 2 h prior to recruitment. Lifelong aspirin (100 mg/day) was prescribed to all patients. At least 12 months of clopidogrel (75 mg/day) or ticagrelor (180 mg/day) was recommended to all patients, based on current guidelines. Patients also received medication of statin, beta blockers, angiotensin-converting enzyme inhibitors, or angiotensin receptor blockers.

Relevant demographic variables were collected for all patients, including age, sex, body mass index, smoking status, alcohol drinking status, and history of hypertension, diabetes mellitus, coronary heart disease, cerebral infarction, and dyslipidemia. Venous blood samples were obtained from peripheral veins in the morning; each included patient had been requested to fast for at least 12 h beforehand. The results of laboratory examinations, including routine white blood cell count, neutrophil count, lymphocyte count, hemoglobin concentration, serum creatinine concentration, serum uric acid concentration, blood lipid levels (total cholesterol, triglycerides, low-density lipoprotein cholesterol (LDL-C), and high-density lipoprotein cholesterol (HDL-C)), and levels of fasting blood glucose, glycosylated hemoglobin, lactate dehydrogenase, creatine kinase, creatine kinase-MB, hypersensitive troponin T, and N-terminal probrain natriuretic peptide (NT-proBNP) were also routinely measured in this study. The serum bile acid level was determined using an enzyme-linked immunosorbent assay; other biomarkers, such as high-sensitive C-reactive protein (hs-CRP), were measured simultaneously.

### 2.3. Study Outcomes and Definitions

After discharge, participants were followed up through outpatient visits, telephone calls, and revised clinical reports and electronic medical records. Follow-up sessions were held to observe whether AMI patients had MACEs, which were defined as a composite of cardiac death, recurrent AMI, unplanned revascularization, or rehospitalization for any cardiovascular disease, including heart failure, nonfatal ischemic stroke, or unstable angina [11].

### 2.4. Statistical Analysis

Continuous variables are reported as means and standard deviations (mean ± SD) or median (25th to 75th percentile) and categorical data are presented as numbers and percentages. Univariate and multivariate logistic regression methods were used to determine predictors of MACEs in AMI patients treated with PCI. Variables that had *P* < 0.05 on univariate analysis were included in the multiple regression analysis. A nomogram was established, based on the results of independent risk factors on multivariable analyses. The nomogram was then subjected to 1000 bootstrap resamples for internal validation. Discrimination was tested using the concordance index, which is identical to the area under the receiver's operating characteristic curve, and ranges from 0.5 (no predictive power) to 1.0 (perfect prediction). The nomogram was calibrated by comparing the predicted probability of MACEs with the actual probability after bias correction with the Hosmer–Lemeshow test. In addition, the clinical usefulness and net benefits of the developed nomogram were assessed using decision curve analysis. The decision curve estimates the net benefit of a model as the difference between the true-positive and false-positive rates, weighted by the odds of the selected threshold probability of risk [12].

## 3. Results

### 3.1. Baseline Characteristics

A total of 2564 AMI patients undergoing PCI in our institution between January 2015 and December 2020 were screened for inclusion. After screening, 1958 AMI patients who had undergone successful intervention of the occluded coronary artery were finally analyzed in the study. Among these, 421 patients developed new-onset MACEs. A total of 561 AMI patients who received PCI in the same center and who were free from MACEs at 1 year after their index admission were enrolled as matched control subjects.  Table 1 summarizes the demographic and biochemical characteristics of AMI patients undergoing PCI, with or without MACEs during follow-up. At baseline, individuals who had had MACEs were significantly older and had a higher proportion of diabetes mellitus, a lower left ventricular ejection fraction (LVEF), and a higher Syntax score than those who did not develop MACEs (all *P* < 0.05). In addition, the serum levels of total cholesterol, LDL-C, lipoprotein (a) (LP(a)), NT-proBNP, bile acid level, and hypersensitive troponin T (hsTnT) were significantly higher (all *P* < 0.05) in patients with MACEs than those without. Nonetheless, there were no significant differences in sex, body mass index, smoking, hypertension, lactate dehydrogenase, creatine kinase, creatine kinase-MB, or hs-CRP between patients with or without MACEs (all *P* > 0.05).

### 3.2. Identification of Risk Factors and Multivariate Analysis for MACEs within 1 Year

As shown in Table 2, univariate and multivariate logistic regression analyses were used to determine the risk factors for MACEs. In the univariate analysis, the involved factors were significantly composed of clinical characteristics, lipid profile, and serum biomarker levels. Next, we entered the predictors with *P* < 0.05 on univariate analysis into the multivariate regression model. In the multivariate logistic regression analysis, based on the results of the univariate and multivariate analyses, independent risk factors for MACEs within 1 year were identified as age (odds ratio (OR): 1.030; 95% confidence interval (CI): 1.014–1.047), diabetes mellitus (OR: 1.667; 95% CI: 1.151–2.415), low-density lipoprotein cholesterol (OR: 1.332; 95% CI: 1.134–1.565), uric acid (OR: 1.003; 95% CI: 1.001–1.005), lipoprotein (a) (OR: 1.003; 95% CI: 1.002–1.003), left ventricular ejection fraction (OR: 0.929; 95% CI: 0.905–0.954), Syntax score (OR: 1.075; 95% CI: 1.053–1.097), and hypersensitive troponin T (OR: 1.002; 95% CI: 1.002–1.003).

### 3.3. Nomogram for Predicting MACEs within 1 Year

**Figure 1:**
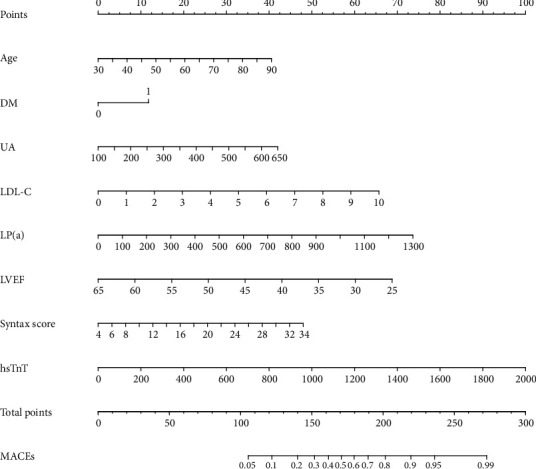
The visible nomogram for predicting 1-year occurrence of MACEs among AMI patients who had PCI. Abbreviations: MACEs: major adverse cardiovascular events; PCI: percutaneous transluminal coronary intervention; AMI: acute myocardial infarction; DM: diabetes mellitus; UA: uric acid; LDL-C: low-density lipoprotein-cholesterol; LP(a): lipoprotein (a); LVEF: left ventricular ejection fraction; hsTnT: hypersensitive troponin T.

After identification of the risk factors for MACEs within 1 year in AMI patients with PCI, the nomogram was constructed, as shown in Figure 1. For each patient, points were assigned for each of identified risk factor or clinical characteristic (age, diabetes mellitus, LDL-C level, LP(a) level, LVEF, Syntax score, and hypersensitive troponin T). A total score calculated from the nomogram corresponded visually to a predictive value for MACEs within 1 year.

### 3.4. Validation of the Nomogram

**Figure 2:**
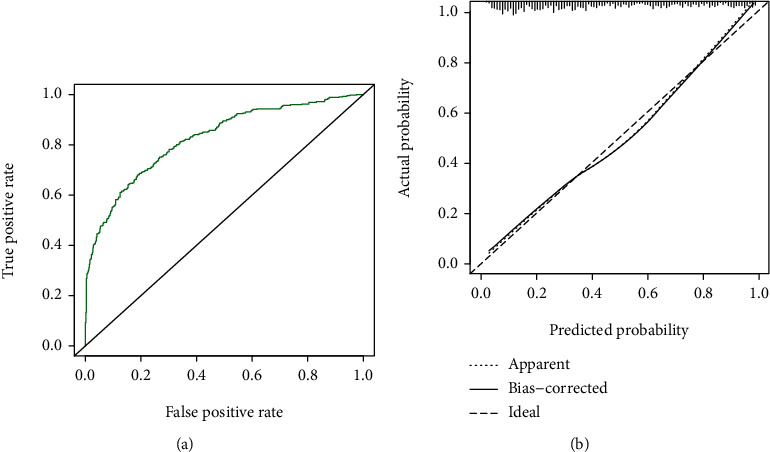
(a) ROC curve of the nomogram for predicting MACEs after PCI in AMI patients. (b) Calibration curve of the nomogram for the prediction model. The *x*-axis represents the overall predicted probability of revascularization after PCI, and the *y*-axis represents the actual probability. Model calibration is indicated by the degree of fitting of the curve and the diagonal. Abbreviations: AUC: area under the ROC curve; ROC: receiver operating characteristic; MACEs: major adverse cardiovascular events; PCI: percutaneous transluminal coronary intervention; AMI: acute myocardial infarction.

**Figure 3:**
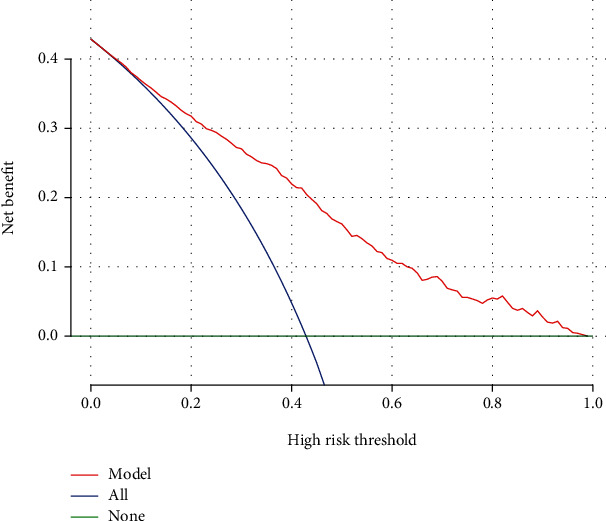
Decision curve analysis for the prediction model. A horizontal line indicates that all samples are negative and not treated, with a net benefit of zero. An oblique line indicates that all samples are positive. The net benefit has a negative slope.

The prediction model showed a *C* statistic of 0.814 (*P* < 0.001), which showed optimal discrimination (Figure 2(a)). In addition, the calibration curve demonstrated good concordance between the predicted and actual outcomes (Figure 2(b)). Likewise, use of the Hosmer–Lemeshow test revealed good calibration of the logistic regression model, indicating that our score model could effectively predict the rate of MACEs within 1 year. To evaluate the clinical validity of the nomogram, decision curve analysis was used to ensure that this model could provide great net benefit (Figure 3). The results of the decision curve analysis indicated that our nomogram was superior to a risk model based on traditional coronary artery disease risk factors alone. The decision curve analysis demonstrated that the nomogram is clinically useful because the ranges of threshold probabilities were wide and practical for the cohort.

## 4. Discussion

The aim of this study was to provide tools for use in predicting 1-year clinical outcomes based on clinical parameters and cardiovascular risk factors in AMI patients after PCI. Our findings first revealed, by multivariate analysis, that AMI patients after stent placement with advanced age, a history of diabetes mellitus, higher baseline serum levels of LDL-C, uric acid, LP(a), and hypersensitive troponin T, higher Syntax scores, and lower LVEFs were more likely to present with MACEs within 1 year. All these variables were integrated in our study to construct a nomogram to predict 1-year MACEs in AMI patients after PCI. The nomogram showed good discrimination (*C* statistic of 0.806) and good calibration. The decision analysis curve demonstrated satisfactory positive net benefits.

It has been demonstrated that traditional risk factors (including advanced age, history of diabetes mellitus, higher baseline serum level of LDL-C, uric acid, and LP(a), higher Syntax score, and lower LVEF) are correlated with the development of MACEs in AMI patients [13–17]. Human functions and physiological processes degrade to different degrees as we age, and age is more likely to be linked with a range of other illnesses. Furthermore, the coronary arteries of older individuals are often accompanied with widespread vascular infections and significant calcification, reducing the likelihood of their getting vascular repair [18]. In the development of coronary artery disease, myocardial infarction, and cardiac death, diabetes mellitus is an independent risk factor. In another study, diabetes mellitus was also identified as the sole independent predictor of repeat hospitalization related to MACEs [17]. Xiong et al. [19] revealed that MACEs and long-term mortality were greater for individuals with diabetes mellitus who were admitted to hospital for acute coronary syndrome. In patients with diabetes mellitus, the risk of MACEs may be greater than that in patients without diabetes mellitus, especially during a follow-up time of less than 3 years, after successful chronic total occlusion PCI in the drug-eluting stent era [20]. However, Yang et al. [21] indicated that strict glycemic control increases the incidence of MACEs in patients with type 2 diabetes mellitus and an acute coronary syndrome who have PCI.

Concentrations of HDL-C less than 40 mg/dL and of LDL-C greater than or equal to 100 mg/dL have been significantly more strongly associated with MACEs in female patients [22]. In addition, MACEs have been linked with cumulative LDL-C exposure in Japanese patients with familial hypercholesterolemia [23]. Moreover, Huang et al. [24] revealed that elevated serum LDL-C levels in patients with type 2 diabetes mellitus might worsen coronary heart disease and predict future cardiovascular events. After further adjustment for the usual risk variables, patients with acute coronary syndrome and hyperuricemia have been found to have a higher risk of MACEs, all-cause mortality, and cardiovascular mortality [25]. Sang et al. [26] have found that the increased risk of hard coronary heart disease, MACE, all-factor mortality, and heart death in patients with acute coronary syndrome are related with high LP(a) levels in patients of advanced age. The Syntax score [27] is a complete angiographic grading system and was developed to assess the severity and extent of coronary stenotic lesions. There was found to be a substantially increased occurrence of stent thrombosis, severe adverse cardiac events, stent revascularization, and repeat revascularization after a high Syntax score [28]. In addition, in those patients with a high Syntax score II, the risk of death was greater following PCI [29]. Left ventricular ejection fraction is a reliable reference measure of cardiac function that is helpful in predicting future health and in determining risk following a myocardial infarction [16]. Our model shows that high hsTnT level is a significant prognostic factor of MACEs and that the MACEs rate increases significantly with an increase in troponin level. Myocilin is a secreted glycoprotein composed of cardiac troponin T, cardiac troponin I, and cardiac troponin C, and elevated troponin level has become a reference index in the diagnosis of AMI [30].

This study is the first clinically validated prediction model in a Chinese population for predicting MACEs after PCI in patients with AMI. Our study found that the serum total bile acid concentration is significant in univariate analysis (*P* = 0.01), but it is not statistically significant compared with the influence of other factors in multivariate analysis (*P* = 0.058). Nevertheless, the serum total bile acid concentration might have some value in predicting the risk of MACE in AMI patients within 1 year after PCI; this can be further explored in later studies. Serum total bile acid concentration is the main metabolite of cholesterol in liver decomposition and the main component of bile and participates in the digestion and absorption of fat. In previous studies, it has gradually been discovered that bile acid level can lead to various types of arrhythmia, such as sinus bradycardia, atrial fibrillation [31], and atrioventricular block, and that it can even lead to the occurrence of cardiac arrest in severe cases, while excessive concentrations of bile acid can cause cardiomyopathy and metabolic dysfunctions in the heart [32]. Many studies have showed that bile acid is associated with atherosclerosis [33, 34] and other cardiovascular diseases, but the underlying mechanisms are not entirely understood. There may be several reasons. Firstly, bile acid is an endogenous inhibitor of 11*β*-hydroxysteroid dehydrogenase; reduced 11*β*-hydroxysteroid dehydrogenase activity and genetic defects both promote salt retention and hypertension [35, 36]. Secondly, as is well known, hypercholesterolemia contributes to the development of atherosclerosis and is a major risk factor for cardiovascular disease. However, enterohepatic circulation of bile acids can maintain cholesterol homeostasis and promote the excretion of excess cholesterol from the body [37].

To our knowledge, this study is the first to report a nomogram based on data from routine clinical variables to predict 1-year MACEs in AMI patients treated with PCI. The identification of patients at high risk of developing MACEs may enable a better understanding for the prevention or treatment of high-risk AMI. However, there are some limitations in this study. The first limitation of this study is that it relies on retrospective research rather than a large multicenter prospective validation study. Another limitation is the absence of a validation dataset in this study. The next step is to further refine the model through multicenter, external validation.

## 5. Conclusions

Our study identifies age, diabetes mellitus, LDL-C level, uric acid level, LP(a) level, LVEF, Syntax score, and hsTnT level as independent predictors of MACEs. We developed a nomogram to predict the risk of MACEs within 1 year in AMI patients after PCI. To ensure generalizability, this model needs to be externally validated.

## Figures and Tables

**Table 1:** Characteristics of the patients.

Variables	Subjects without MACEs	Subjects with MACEs	*P* value
Age, year	64.12 ± 9.94	67.35 ± 10.02	<0.001
Gender (*n*, %)			0.538
Male	216 (38.50%)	154 (36.58%)	
Female	345 (61.50%)	267 (63.42%)	
Smoking (*n*, %)			0.334
No	337 (60.07%)	240 (57.01%)	
Yes	224 (39.93%)	181 (42.99%)	
Drinking (*n*, %)			0.362
No	283 (50.45%)	200 (47.51%)	
Yes	278 (49.55%)	221 (52.49%)	
Hypertension (*n*, %)			0.979
No	211 (37.61%)	158 (37.53%)	
Yes	350 (62.39%)	263 (62.47%)	
Diabetes mellitus (*n*, %)			0.003
No	452 (80.57%)	305 (72.45%)	
Yes	109 (19.43%)	116 (27.55%)	
History of CHD (*n*, %)			0.854
No	555 (98.93%)	417 (99.05%)	
Yes	6 (1.07%)	4 (0.95%)	
History of cerebral infarction (*n*, %)			0.244
No	499 (88.95%)	384 (91.21%)	
Yes	62 (11.05%)	37 (8.79%)	
History of dyslipidemia (*n*, %)			0.773
No	559 (99.64%)	419 (99.52%)	
Yes	2 (0.36%)	2 (0.48%)	
AMI classification (*n*, %)			0.46
STEMI	328 (58.47%)	256 (60.81%)	
NSTEMI	233 (41.53%)	165 (39.19%)	
BMI (kg/m^2^)	23.27 ± 5.18	23.01 ± 5.22	0.554
WBC (×10^9^/L)	7.06 ± 2.26	6.88 ± 1.99	0.348
Hemoglobin	138.23 ± 43.99	136.13 ± 16.12	0.814
Neutrophil granulocyte (×10^9^/L)	4.61 ± 2.09	4.45 ± 1.87	0.321
Lymphocyte (×10^9^/L)	1.84 ± 0.63	1.84 ± 0.63	0.860
hs-CRP (mg/L)	51.17 ± 28.26	51.41 ± 28.44	0.908
FBG (mmol/L)	6.33 ± 2.16	6.55 ± 2.28	0.201
Glycosylated hemoglobin (%)	6.62 ± 1.18	6.64 ± 1.41	0.881
Total cholesterol (mmol/L)	4.69 ± 1.15	4.79 ± 1.28	0.585
Triglycerides (mmol/L)	1.68 ± 1.01	1.70 ± 1.11	0.767
HDL-C (mmol/L)	1.25 ± 0.33	1.28 ± 0.33	0.361
LDL-C (mmol/L)	2.59 ± 0.99	2.87 ± 0.92	<0.001
LP(a) (mg/L)	196.47 ± 187.95	306.99 ± 228.96	<0.001
Uric acid (*μ*mol/L)	291.04 ± 78.95	312.05 ± 81.58	<0.001
Creatinine (*μ*mol/L)	67.61 ± 17.93	70.27 ± 21.90	0.011
LDH (U/L)	510.16 ± 106.39	514.11 ± 103.16	0.533
CK (U/L)	1919.71 ± 959.49	1996.05 ± 970.02	0. 206
CK-MB (ng/mL)	243.12 ± 117.91	254.06 ± 109.72	0.192
hsTnT (ng/L)	676.28 ± 317.03	1008.12 ± 461.73	<0.001
NT-proBNP (pg/mL)	692.97 ± 237.12	737.62 ± 222.82	0.006
LVEF (%)	49.87 ± 5.85	46.75 ± 6.55	<0.001
Syntax score	14.68 ± 6.93	18.98 ± 8.72	<0.001
DAPT (*n*, %)			0.193
No	33 (5.88%)	17 (4.04%)	
Yes	528 (94.12%)	404 (95.96%)	
ACE inhibitor (*n*, %)			0.365
No	32 (5.70%)	30 (7.13%)	
Yes	529 (94.30%)	391 (92.87%)	
Beta blocker (*n*, %)			0.731
No	33 (5.88%)	27 (6.41%)	
Yes	528 (94.12%)	394 (93.59%)	
Statin (*n*, %)			0.621
No	33 (5.88%)	28 (6.65%)	
Yes	528 (94.12%)	393 (93.35%)	
Bile acid level (*μ*mol/L)	6.87 ± 2.37	7.26 ± 2.24	0.008

Abbreviations: MACEs: major adverse cardiovascular events; CHD: coronary heart disease; BMI: body mass index; WBC: white blood cell; FBG: fasting blood glucose; HDL-C: high-density lipoprotein-cholesterol; LDL-C: low-density lipoprotein-cholesterol; LP(a): lipoprotein (a); WBC: white blood cell; LDH: lactate dehydrogenase; CK: creatine kinase; LVEF: left ventricular ejection fraction; CK-MB: creatine kinase-MB; hsTnT: hypersensitive troponin T; NT-proBNP: N-terminal probrain natriuretic peptide; ACE: angiotensin-converting enzyme; DAPT: dual antiplatelet therapy; Syntax: synergy between percutaneous coronary intervention.

**Table 2:** Univariate and multivariate logistic regression analyses for 1-year MACEs.

Variables	Univariate analysis	*P* value	Multivariate analysis	*P* value
	OR (95% CI)		OR (95% CI)	
Age, years	1.033 (1.020, 1.047)	<0.001	1.030 (1.014, 1.047)	<0.001
DM, no vs. yes	1.577 (1.167, 2.128)	0.003	1.667 (1.151, 2.415)	0.007
Creatinine (*μ*mol/L)	1.007 (1.000, 1.014)	0.039	1.003 (0.994, 1.001)	0.539
Uric acid (*μ*mol/L)	1.003 (1.002, 1.005)	<0.001	1.003 (1.001, 1.005)	0.001
LDL-C (mmol/L)	1.356 (1.861, 1.556)	<0.001	1.332 (1.134, 1.565)	<0.001
LP(a) (mg/L)	1.002 (1.002, 1.003)	<0.001	1.003 (1.002, 1.003)	<0.001
NT-proBNP (pg/mL)	1.0008 (1.0003, 1.0014)	0.002	1.000 (1.000, 1.001)	0.233
LVEF (%)	0.921 (0.901, 0.941)	<0.001	0.929 (0.905, 0.954)	<0.001
Syntax score	1.071 (1.054, 1.090)	<0.001	1.075 (1.053, 1.097)	<0.001
hsTnT (ng/L)	1.002 (1.002, 1.003)	<0.001	1.002 (1.002, 1.003)	<0.001
Bile acid level (*μ*mol/L)	1.074 (1.017, 1.135)	0.01	1.067 (0.998, 1.142)	0.058

Abbreviations: MACEs: major adverse cardiovascular events; DM: diabetes mellitus; LDL-C: low-density lipoprotein-cholesterol; LP(a): lipoprotein-a; LVEF: left ventricular ejection fraction; hsTnT: hypersensitive troponin T; NT-proBNP: N-terminal probrain natriuretic peptide; Syntax: synergy between percutaneous coronary intervention.

## Data Availability

The raw data supporting the conclusions of this article will be made available by the authors, without undue reservation.

## Supplementary Materials

Supplementary Materials Supplementary File S1: inclusion and exclusion criteria of participants. Supplementary File S2: informed consent and questionnaire from the Affiliated Hospital of Xuzhou Medical University.
