# Supplementary material for: Clinical Nomogram to Predict Major Adverse Cardiac Events in Acute Myocardial Infarction Patients within 1 Year of Percutaneous Coronary Intervention
Source: Cardiovasc Ther. 2021 Dec 13;2021:3758320. doi: 10.1155/2021/3758320 (PMC8687843; doi:10.1155/2021/3758320)
Supplement: Supplementary Materials — Supplementary File S1: inclusion and exclusion criteria of participants. Supplementary File S2: informed consent and questionnaire from the Affiliated Hospital of Xuzhou Medical University. [file 3758320.f1.zip › Supplementary File S1.docx]

**Supplementary File S1. Inclusion and exclusion criteria of participants.**

**Inclusion criteria** **of patients:**

(1) patients were diagnosed with either ST-segment elevation MI (STEMI) or non-STEMI(NSTEMI);

(2) the coronary angiography showed that patients have received PCI for anatomy in infarct-related artery;

(3) the follow-up time was at least 12 months.

**Exclusion criteria of acute myocardial infarction:**

Individuals with a history of congenital heart disease, rheumatic heart disease, viral myocarditis, chronic obstructive pulmonary disease, peripheral vascular diseases, recent history of infection or trauma, cancer, autoimmune disorders, abnormal renal/liver function, hemopathy or thyroid dysfunction were excluded.
