# Supplementary material for: Clinical Nomogram to Predict Major Adverse Cardiac Events in Acute Myocardial Infarction Patients within 1 Year of Percutaneous Coronary Intervention
Source: Cardiovasc Ther. 2021 Dec 13;2021:3758320. doi: 10.1155/2021/3758320 (PMC8687843; doi:10.1155/2021/3758320)
Supplement: Supplementary Materials — Supplementary File S1: inclusion and exclusion criteria of participants. Supplementary File S2: informed consent and questionnaire from the Affiliated Hospital of Xuzhou Medical University. [file 3758320.f1.zip › Supplementary material legends.docx]

**Supplementary material legends**

**Supplementary File S1.** Inclusion and exclusion criteria of participants.

**Supplementary File S2.** Informed consent and questionnaire from the Affiliated Hospital of Xuzhou Medical University.
