# Supplementary material for: Clinical Nomogram to Predict Major Adverse Cardiac Events in Acute Myocardial Infarction Patients within 1 Year of Percutaneous Coronary Intervention
Source: Cardiovasc Ther. 2021 Dec 13;2021:3758320. doi: 10.1155/2021/3758320 (PMC8687843; doi:10.1155/2021/3758320)
Supplement: Supplementary Materials — Supplementary File S1: inclusion and exclusion criteria of participants. Supplementary File S2: informed consent and questionnaire from the Affiliated Hospital of Xuzhou Medical University. [file 3758320.f1.zip › Supplementary File S2.doc]

Informed consent and questionnaire

**from the Affiliated Hospital of Xuzhou Medical University**

**Dear Participants:**

We warmly invite you to participate in a research project “Clinical nomogram based on the clinical parameters and serum bile acid level to predict major adverse cardiac events in acute myocardial infarction patients after percutaneous coronary intervention within one year” approved by the ethics committee of the Affiliated Hospital of Xuzhou Medical University. This study will be conducted in the Affiliated Hospital of Xuzhou Medical University, and it is estimated that 400 subjects will voluntarily participate.

You have abundant rights to decide whether participate our study. Even if you have signed your consent to participate, you can change your decision at any stage of the study to abandon participation. No reason is needed for withdrawing from the study and we fully respect your autonomy.

**Why was this study conducted?**

It is widely recognized that acute myocardial infarction (AMI) is the leading cause of morbidity and mortality worldwide. Numerous studies have revealed that primary percutaneous coronary intervention (PCI) can increase myocardial blood flow perfusion and improve clinical prognosis. However, AMI patients undergoing PCI still have an increased risk of recurrent major adverse cardiovascular events (MACEs). The reason is that the pathogenesis of AMI is complex. Although previous researches from western country patients reported that diabetes, current smoking, history of heart failure, and high syntax score were independent prognostic factors of MACEs for AMI patients treated with PCI. However, few researches have explored the cardiovascular risk stratification and outcome prediction in Chinese AMI patients after successfully PCI. Nomograms are widely used to predict disease prognosis for an individual patient. Thus, our study aimed to establish a prognostic nomogram based on the clinical parameters and serum bile acid level for use in the Chinese patients that can help clinicians to both identify patients who are at high-risk and provide them proper treatment by more accurate risk estimation.

**Who should not be included in the study?**

If you are: I) patients with previous history of heart valve disease, congenital heart disease, cardiomyopathy, chronic heart failure, II) patients with previous stroke, familial hypercholesterolemia, severe hepatorenal pulmonary and thyroid disease, neoplasm, sepsis and patients with familial and hereditary diseases, III) pregnant, complicated by local or systemic infection; IV) mental illness, alcohol addiction, drug abusers.

**What needs to be done if enrolled in the study?**

If you are a patient with STEMI and willing to participate in this study, you will answer and fill in the following questions.

1. Gender：□Male □Female
2. Age_______（year）
3. Height_______（cm）
4. Weight_______（kg）
5. History of surgery：□No □Yes（______years ago, term of operation：_________）

Receiving blood products：□No □Yes

6. Smoke：□No □Yes（______years, ______branches/day，forbid smoking for approximately ______year）
7. Drink：□No □Yes（Category □Liquor and spirits □Wine □Beer，____year, ____g/day，frobid drinking for approximately ____days）
8. Physical activity：□Normal □Restricted
9. Hypertension：□No □Unclear □Yes（Highest___/___mmHg, Lowest____/___mmHg; Feel dizzy when blood pressure up to ______mmHg，Normal blood pressure _______ mmHg）
10. Diabetes mellitus：□No □Unclear □Yes（□Drug □Insulin）
11. History of coronary heart disease：□No □Yes
12. Dietary：□Normal □Over □Less □Unable to eat
13. History of gastric or duodenal ulcers：□No □Unclear □Yes
14. History of drug allergy：□No □Yes（Name___________）
15. History of food allergy：□No □Yes（Name______________）
16. The recent medication：□No □Yes（Name_____________）
17. Menstruation：□Menstrual period □Non-menstrual period
18. Relatives (blood relationship) related diseases:-without/with _____________________ (name)_____________________）

**What are the benefits of participating in the study?**

We developed and internally validated a visible nomogram to predict the risk of MACEs within 1 year in AMI patients after PCI.

**Is there a fee to pay for participating in the study?**

You are not required to pay for this, and if a study related adverse reaction occurs, we will take steps to prevent it.

**Do I have to take part in the study?**

Participation in this study is completely voluntary and you may refuse to participate in the study, or withdraw from the study at any time during the course of the study, which will not affect your treatment by your doctor. If you decide to withdraw from this study, please contact your doctor and you may be asked for the relevant tests, which are beneficial for protecting your health.

If you believe that study damage has occurred, please contact your study doctor at the following contact number: 13852438611.

If you feel your interests are compromised, please contact the hospital ethics committee at: 0516-85806993.

Subject statement: I have read the above introduction to this study, being well informed of the risks and benefits that may result from participation in this study.

I volunteered to participate in this study. I agree □ decline □ other studies than this study utilize my medical records and specimens for pathologic examination.

Subject's signature: Date:

Subject's contact phone: Mobile phone:

Doctor states: I confirm that details of this study have been explained to the patient, in particular the risks and benefits that may result from participating in this study.

Physician's signature: Date:

Physician's working phone: Mobile phone:

Clinical trial ethics committee of Affiliated Hospital of Xuzhou Medical University contact number: 0516-85802291.
